# Identification of commonly expressed exoproteins and proteolytic cleavage events by proteomic mining of clinically relevant UK isolates of *Staphylococcus aureus*

**DOI:** 10.1099/mgen.0.000049

**Published:** 2016-02-23

**Authors:** Debra S. Smith, Matthew K. Siggins, Magdalena Gierula, Bruno Pichon, Claire E. Turner, Nicola N. Lynskey, Mia Mosavie, Angela M. Kearns, Robert J. Edwards, Shiranee Sriskandan

**Affiliations:** ^1^​ Department of Medicine, Imperial College London, Hammersmith Hospital Campus, Du Cane Road, London W12 0NN, United Kingdom; ^2^​ Antimicrobial Resistance and Healthcare Associated Infections Reference Unit, National Infection Service, Public Health England, 61 Colindale Avenue, London, NW9 5EQ, United Kingdom

**Keywords:** MS, proteolysis, SasG, *Staphylococcus aureus*

## Abstract

The range of exoproteins and core exoproteome of 14 *Staphylococcus aureus* isolates representing major lineages associated with asymptomatic carriage and clinical disease in the UK was identified by MS proteomics using a combined database incorporating sequences derived from 39 *S. aureus* genomes. In all, 632 different proteins were identified and, of these, only 52 (8 %) were found in all 14 isolates whereas 144 (23 %) were found in just a single isolate. Comparison of the observed mass of each protein (based on migration by SDS-PAGE) with its predicted mass (based on amino acid sequence) suggested that 95 % of the proteins identified were not subject to any major post-translational modification. Migration of 5 % of the proteins was not as expected: 1 % of the proteins migrated at a mass greater than predicted, while 4 % appeared to have undergone proteolytic cleavage; these included SsaA2, Aur, SspP, Ebh as well as BlaR1, MecR1, FsH, OatA and LtaS. Intriguingly, a truncated SasG was produced by a single CC8 USA300-like strain. The analysis provided evidence of the marked heterogeneity in protein expression by *S. aureus* in broth, while yielding a core but narrow common exoproteome.

## Data Summary

Tables S1–S3 have been deposited in figshare. DOI: http://dx.doi.org/10.6084/m9.figshare.1566807

Table S4 (text file), non-overlapping combined database used to identify *S. aureus* proteins, has been deposited in figshare. DOI: http://dx.doi.org/10.6084/m9.figshare.1566710

Genome data from the 14 isolates used are deposited in the European Nucleotide Archive (http://www.ebi.ac.uk/ena, accession number PRJEB12240, secondary study accession number ERP013694).

## Impact Statement

Proteins secreted by 14 strains from clinically relevant major lineages of *S. aureus* were identified using an unbiased proteomic method that made no prior assumptions as to the perceived importance, class or location of exoproteins. Surprisingly, out of over 600 different proteins found, only 8 % were common to all lineages, underlining the extreme heterogeneity of the *S. aureus* exoproteome, with relevance for both development of microbial diagnostics and pathogenetic studies in this species. Intriguingly, the approach simultaneously identified novel proteolytic events and hitherto unsuspected truncated proteins that may impact on virulence and pathogenesis of *S. aureus*.

## Introduction

*Staphylococcus aureus* is a major nosocomial and community-acquired pathogen, which is carried asymptomatically by much of the population in the anterior nares either persistently or intermittently (Vandenbergh & Verbrugh, 1999). Often the cause of minor skin infections, it can give rise to systemic infections affecting the blood, bone, heart or lung and trigger toxin-mediated disease such as toxic shock syndrome, while meticillin-resistant *S. aureus* (MRSA) poses additional management challenges (Rudkin *et al.*, 2012).

Since publication of the N315 and Mu50 genomes by Kuroda *et al.* (2001), the number of sequenced *S. aureus* genomes has increased rapidly. Currently, about 50 completed *S. aureus* genomes have been deposited at NCBI, whilst over 600 projects are in progress (www.ncbi.nlm.nih.gov/genome/genomes/154). Data from such whole genome sequencing projects have demonstrated that there is a high level of diversity within the species, with variability occurring in approximately 20–30 % of the genome (Witney *et al.*, 2005). Approximately 70 % of the genes can be considered core (present in >95 % isolates), while 10–12 % comprise ‘core-variable’ genes that are lineage specific; the remainder are encoded by mobile genetic elements and comprise the variable genome (Lindsay *et al.*, 2006). Although some virulence genes reside within the core genome, the vast majority of core genes encode proteins with housekeeping functions. Conversely, most of the variable and core variable genes are involved in the interaction of the bacterium with its environment, its host or other bacterial cells.

Sibbald *et al.* (2006) defined the core exoproteome of *S. aureus* as the 58 proteins with predicted Sec-type signal peptides encoded by genes present in all sequenced *S. aureus* strains. While this *in silico* bioinformatic approach is highly efficient, it cannot provide data on expression, post-translational modifications, cleavage and turnover, which may be important considerations in pathogenesis research or biomarker studies. Furthermore, as noted by Sibbald *et al.*, and many others, Sec-secreted proteins are only a subset of the proteins found in the supernatants of *S. aureus* cultures (Henderson & Martin, 2011; Sibbald *et al.*, 2006; Tjalsma *et al.*, 2004). Many groups have attempted to define the proteome of *S. aureus* (Becher *et al.*, 2009; Nandakumar *et al.*, 2005; Ravipaty & Reilly, 2010; Sibbald *et al.*, 2006; Ziebandt *et al.*, 2010), to identify vaccine candidates (Glowalla *et al.*, 2009; Vytvytska *et al.*, 2002), to study virulence factors and their regulators (Bernardo *et al.*, 2002; Burlak *et al.*, 2007; Kawano *et al.*, 2001; Nakano *et al.*, 2002; Pocsfalvi *et al.*, 2008; Rogasch *et al.*, 2006; Ziebandt *et al.*, 2001, 2004), and to study changes in proteins in response to different conditions such as anaerobiosis (Fuchs *et al.*, 2007) and glucose starvation (Michalik *et al.*, 2009).

In this study we set out to determine the core exoproteome of 14 clinical *S. aureus* isolates representing the dominant clinical lineages identified in the UK. Our approach was not biased towards any particular protein, based on virulence or any other phenomenon, and made no assumptions about proteins that would or would not be secreted by the bacterium. We analysed *S. aureus* culture supernatant proteins using a GeLC-MS proteomic approach and identified the proteins using a database that combined sequences from 39 *S. aureus* completed genomes to identify as many proteins as possible amongst the clinical isolates studied, in a single proteomic study.

## Methods

### Bacterial strains and growth conditions

Fourteen temporally and geographically unrelated isolates of *S. aureus* [six meticillin-susceptible *S. aureus* (MSSA), eight MRSA] were selected to represent a broad spectrum of disease and genetic diversity (including 11 different MLST clonal complexes). Isolates were grown with shaking at 37 °C in Lysogeny broth (LB), tryptic soy broth, casein hydrolysate-yeast extract-containing medium, RPMI and RPMI containing 0.15 mM desferrioxamine (to sequester iron). Bacterial growth was assessed by measuring OD_600_ of appropriately diluted samples of the culture mixture using a photometer (Biophotometer; Eppendorf) on three separate occasions in fresh LB.

### SDS-PAGE

Proteins were precipitated from 3.5 ml of filtered (0.22 μm) culture supernatants obtained by addition of three volumes of 40 % trichloroacetic acid in acetone overnight at − 20 °C; the exact volumes were adjusted based on OD_600_ measurements to normalize protein loading on this basis. Protein pellets were washed twice with acetone, dried, dissolved in 60 μl LDS electrophoresis sample treatment buffer with 40 mM DTT and heated at 70 °C for 10 min. Iodoacetamide (200 mM) was added to 18 μl of the sample and incubated for 20 min prior to loading onto a 10 % Bistris pre-cast gel (Life Technologies) and separation using MOPS buffer. Gels were stained for protein with InstantBlue (Expedeon).

### Membrane integrity of *S. aureus*

In total, 5 × 10^8^ c.f.u. *S. aureus* HHS-1, -7, -8 and -9 were stained with a final concentration of 30 μM propidium iodide (PI) in 500 μl PBS, incubated protected from light for 5 min and then analysed on a FACSCalibur flow cytometer (BD Biosciences). *S. aureus* was identified by light-scatter characteristics, and PI fluorescence was measured in the FL2 channel. Heat-treated *S. aureus* was incubated at 60 °C for 30 min prior to staining.

### Molecular typing/PCR

Typing of *spa* was carried out as described previously (Harmsen *et al.*, 2003). MLST clonal complex assignments were inferred based on *spa* typing data and by reference to the spa server (http://spa.ridom.de/mlst.shtml). Specific sequence types (STs) were assigned following whole genome sequencing. Selected toxin genes were screened for by multiplex PCR, including: enterotoxins A–E and G–J (*sea–see* and *seg–sej*), toxic shock syndrome toxin-1 (*tst*), exfoliative toxins A, B and D (*eta*, *etb* and *etd*), and Panton–Valentine leukocidin (*luk-PV*), as described previously (Holmes *et al.*, 2005); *sep* and *ser* were detected by genome sequencing. Characterization of staphylococcal cassette chromosome *mec* (SCC*mec*) elements was carried out on all MRSA isolates as described by Milheiriço *et al.* (2007). The *sasG* gene was amplified by PCR from genomic DNA extracted from HHS5 using primers 5′-GTCAAAGATGGGGCC-3′ and 5′-CTTTCGATAATCCTGG-3′.

### Sample preparation for LC-MS/MS

Isolates were grown in LB until they reached exponential phase (OD_600_ of 2.0). Supernatant proteins were precipitated, separated by SDS-PAGE and stained for protein as described above. Each lane in the gel was cut into 27 rows. The proteins present in each gel slice were digested with trypsin, and the resultant peptides were extracted and analysed by LC-MS/MS as described previously (Zhu *et al.*, 2008).

### Protein identification

Identification of the proteins present in each gel slice was determined from analysis of the LC-MS/MS data using sequest and a non-redundant database based on 39 completed *S. aureus* genome sequences available on the PATRIC database (brcdownloads.vbi.vt.edu/patric2/genomes/) as of 28 October 2013. The database comprised 31 784 different protein sequences followed by 20 common contaminants such as human keratins. The sequest software assigns protein designations using the first instance in the database, so the order that the *S. aureus*fasta sequences appear in the database can be of relevance in the listed output of identified proteins. *S. aureus* COL protein sequences were placed first in the reference database, which was then expanded sequentially by addition of non-identical protein sequences that occur in the following strains: NCTC 8325, JH1, MW2, Mu50, N315, Newman, USA300_FPR3757, MSSA476, MRSA252, JH9, Mu3, USA300_TCH1516, JKD6008, ED98, TW20, ST398, 04-02981, ED133, JKD6159, HO 5096 0412, RF122, LGA251, 08BA02176, CA-347, M1, ST228/10388, ST228/10497, ST228/15532, ST228/18412, 11819-97, 55/2053, 71193, ECT-R 2, M013, MSHR1132, T0131, TCH60 and VC40. All sequest results were filtered based on peptide cross correlation scores exceeding 1.5 (single-charged ions), 2.0 (double-charged ions) and 2.5 (triple-charged ions) and identification of at least two different peptides to a protein with a probability score < 0.01. Using a database with fasta sequences from several genomes increased the chance of correctly assigning a protein designation, but also introduced the possibility of peptides from a single protein matching to multiple orthologues and to indicate falsely the presence of several different proteins. To address this, non-COL protein identifications were assigned to an orthologous COL sequence or, in those cases where no COL orthologue was found, to another suitable reference sequence. This was achieved by calculating the minimum Levenstein distance for each non-COL protein identified amongst all protein sequences in the COL database. Non-COL protein sequences with less than 20 % mismatches were assigned to the equivalent COL protein. All others were analysed using blast against a representative set of *S. aureus* genomes (NCTC 8325, JH1, MW2, Mu50, Newman, USA300_FPR3757 and MRSA252) and assigned to their closest orthologue. Where no match was found the protein retained its original assignment. The program psortb was used to identify the predicted cellular location for each protein identified (Yu *et al.*, 2010).

### Analysis of the migration of proteins following SDS-PAGE

The theoretical mass of each protein was calculated from its predicted sequence as occurs in the PATRIC database without taking into account any known or assumed modifications. The actual mass deduced from migration by SDS-PAGE was determined by comparison with the migration of electrophoretic markers (SeeBlue Plus2 Pre-stained Protein Standard; Life Technologies). For proteins identified in a single row the migration position was taken as the centre of the excised row. For those proteins that occurred in multiple contiguous rows, the migration position was estimated as a weighted average based on the frequency of occurrence of the protein in the different isolates.

## Results

### Characterization of the exoproteome of 14 *S. aureus* isolates

**Fig. 1:**
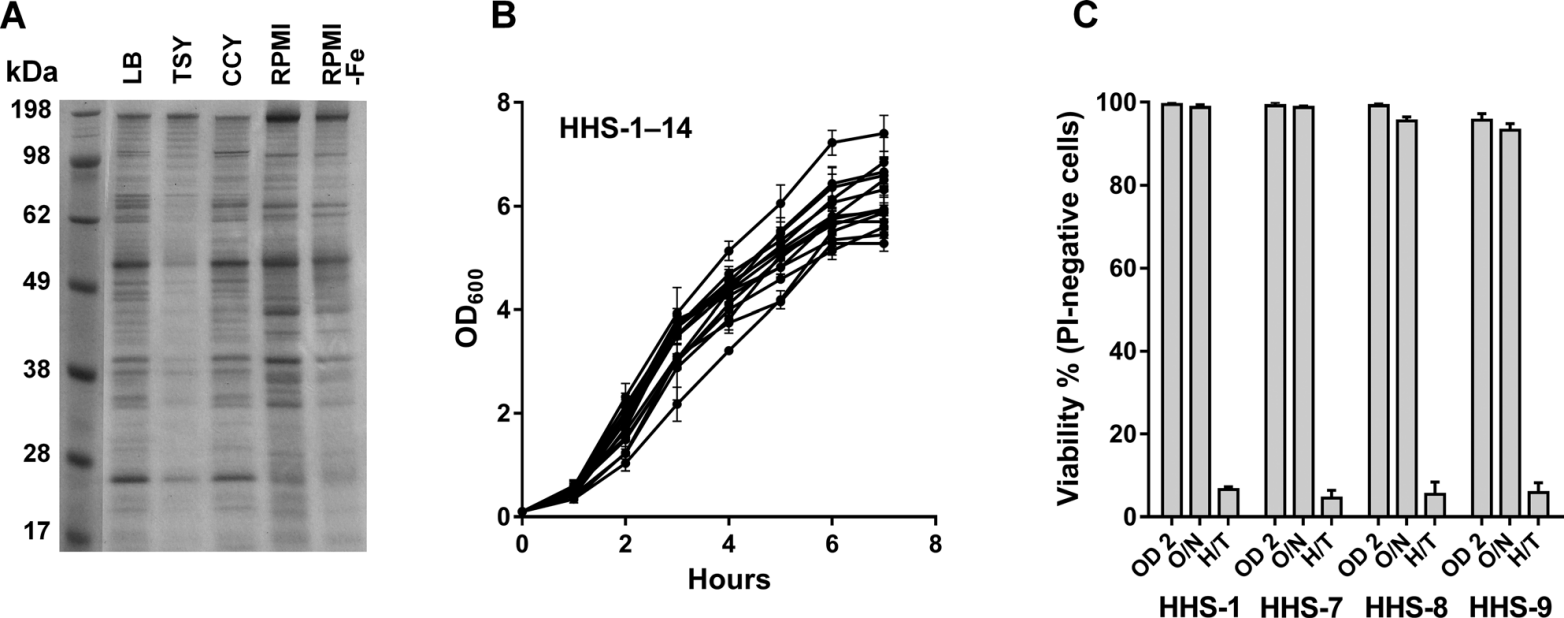
Investigation of culture conditions suitable for proteomic analysis. (a) SDS-PAGE analysis of *S. aureus* isolate HHS-1 cell-free supernatant after culture in LB, tryptic soy broth (TSY), casein hydrolysate-yeast extract-containing (CCY) medium, RPMI and RPMI containing desferrioxamine (RPMI-Fe). Supernatant proteins were precipitated and amounts equivalent to a volume of cells with an OD_600_ of 1.0 were loaded onto the gel. (b) Growth curves showing OD_600_ over time for all 14 *S. aureus* isolates, HHS-1 to HHS-14, in LB; error bars represent sem of three separate LB cultures on different days. (c) Membrane integrity of *S. aureus* isolates HHS-1, -7, -8 and -9 determined by exclusion of PI, measured by flow cytometry, cultured to exponential growth phase, OD_600_ of 2.0 (OD 2), overnight (O/N) and heat-treated (H/T).

The molecular characterization of the study isolates is shown in Table 1. Isolate HHS-1 was grown in five different media and the proteins present in each of the supernatants were analysed by SDS-PAGE (Fig. 1a). Although there was some variation in the overall intensity of staining, the composition of the proteins appeared similar in each case. The result obtained using LB appeared representative and so was selected for use in further studies. Growth characteristics in LB were examined for all the isolates. Each isolate grew rapidly in culture after a short lag phase until an OD_600_ of approximately 5 was reached when growth slowed (Fig. 1b, and Fig. S1, available in the online Supplementary Material). The integrity of the bacterial membrane was assessed in four strains by measuring the proportion of cells capable of excluding PI. When cultured to exponential phase (OD_600_ of 2.0), 99 % of the bacteria excluded PI (isolates HSS-1, HHS-7 and HSS-8) with the exception of HSS-9, where 95 % of the bacteria excluded PI. Bacterial cell membrane integrity was reduced further after culturing overnight (OD_600_ of >6) to 93–99 %, whilst heat treatment of the bacteria for 30 min resulted in substantial loss of ability to exclude PI (∼5 % in each case) (Fig. 1c). Thus, culturing the bacteria to exponential phase growth (OD_600_ of 2.0) appeared appropriate for studies of proteins released by viable cells into the supernatant.

**Table 1:** Characteristics of the 14 *S. aureus* isolates studied -, Not applicable; ST, sequence type derived *in silico* from genome data.

Strain	Year of isolation	Clinical presentation	Toxin gene profile	SCC*mec* type	*spa* type	MLST clonal complex	ST
HHS-1	2006	Bacteraemia	*sea*, *seh*	IV_NT_	t127	1	1
HHS-2	2005	Skin abscess	*sed*, *seg*, *sei*, *sej*, *sep*, *ser*, *luk-PV*	IVc	t002	5	5
HHS-3	2009	Skin abscess	*sed*, *seg*, *sei*, *sej*, *ser*	VI	t002	5	5
HHS-4	2009	Burn	− *	III	t037	8	239
HHS-5	2004	Skin abscess	*luk-PV*	IVa	t008	8	8
HHS-6	2007	Scalded skin syndrome	*seg*, *sei*, *eta*	−	t209	9	109
HHS-7	2006	Bacteraemia	*sec*, *sep*	−	t156	12	1460
HHS-8	2006	Bacteraemia	− *	−	t084	15	15
HHS-9	2006	Bacteraemia	*sec*, *seg*, *sei*	IVh	t032	22	22
HHS-10	2008	Necrotizing pneumonia	*seg*, *sei*, *etd*, *luk-PV*	−	t660	25	25
HHS-11	2006	Bacteraemia	*sea*, *seg*, *sei*, *tst*	II	t018	30	36
HHS-12	2006	Bacteraemia	*seg*, *sei*, *luk-PV*	IVc	t019	30	30
HHS-13	2007	Skin abscess	*seg*, *sei*	−	t015	45	45
HHS-14	2007	Scalded skin syndrome	*seg*, *sei*, *eta*	−	t171	121(51)	1693

* Specified toxin genes not detected.

**Fig. 2:**
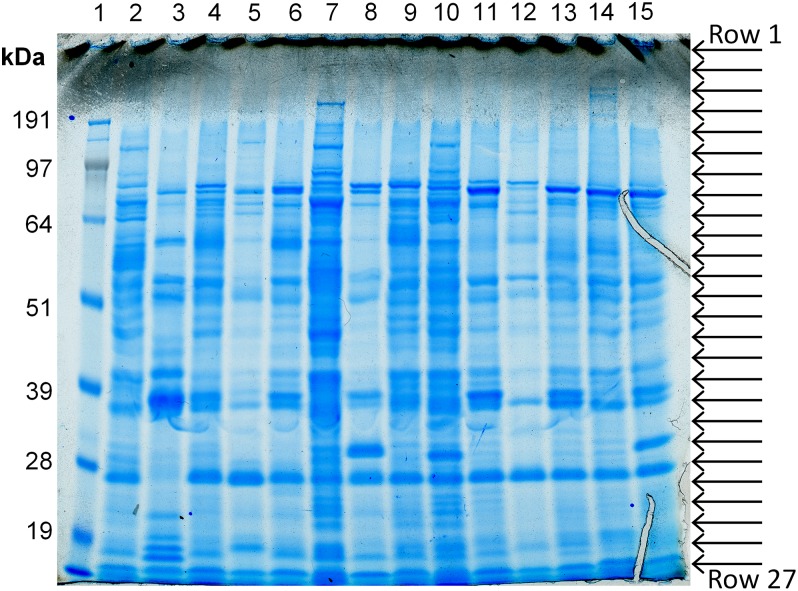
SDS-PAGE of proteins present in the supernatant of 14 *S. aureus* study isolates cultured in LB for proteomic analysis. Supernatant proteins from each of the culture isolates were precipitated and amounts equivalent to a volume of cells in exponential phase growth with an OD_600_ of 2.0 were loaded onto the gel. Arrows indicate approximate positions where gel was sliced into 27 rows. Lane M, marker; lanes 1–14, isolates HHS-1 to HHS-14, respectively.

All 14 isolates were cultured to exponential phase (OD_600_ of 2.0) in LB, and SDS-PAGE of proteins present in each of the culture supernatants was performed (Fig. 2). This showed that although it was evident that some bands occurred in most of or all the supernatant samples, there was also a high degree of variation in both the intensity and the distribution of many individual bands noted (Table 1).

Proteomics was performed on individual gel slices to identify proteins migrating throughout the gel, and the respective mass at which proteins migrated. In all, 632 different proteins were identified amongst the 14 study isolates (Tables S1–S3). Of these, it was possible to assign 569 (90 %) to COL protein sequences using the merged database of 39 genomes (Table S4). The remaining non-COL proteins were mainly linked to mobile genetic elements. The program psortb was used to predict a cellular location for each exoprotein identified with the following results: 5 % cell wall, 8 % cytoplasmic membrane, 64 % cytoplasmic, 10 % extracellular and 13 % unknown.

### Distribution of proteins amongst the isolates

**Fig. 3:**
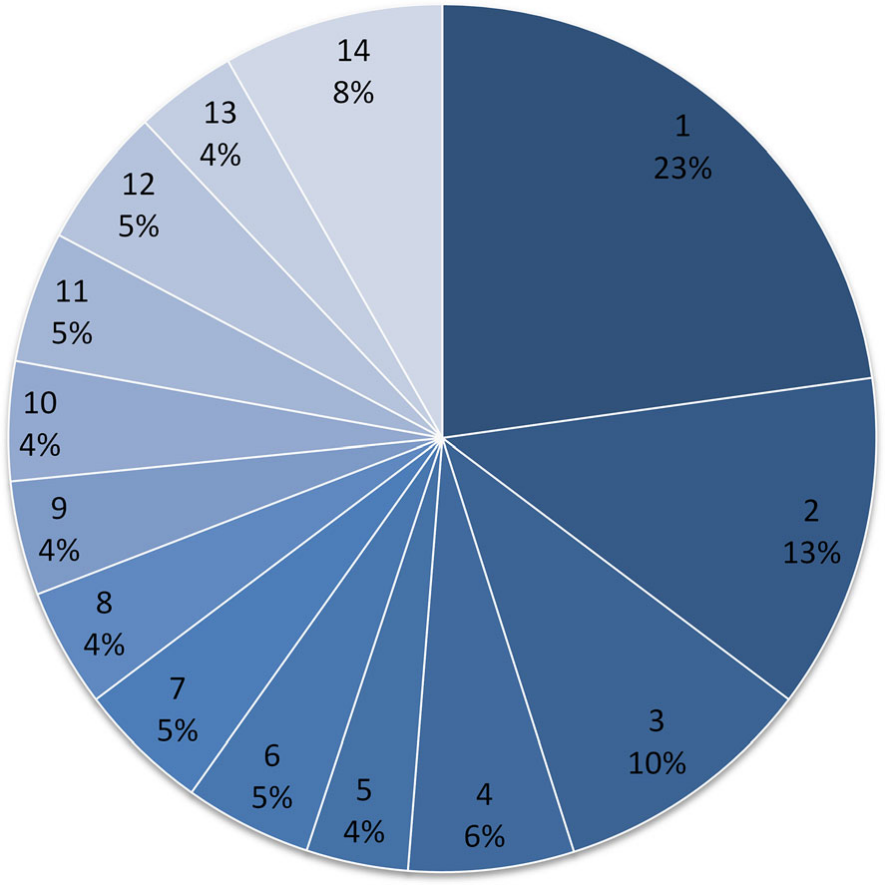
Percentage distribution of *S. aureus* proteins amongst the isolates studied. The occurrence of every protein identified in each of the 14 strains studied was assessed and presented as percentages of the number of proteins found in just one strain (i.e. unique occurrence) or common to between two and 14 strains, with 14 indicating that the proteins were found in all strains studied.

Of the 632 proteins identified, just 52 (8 %) were identified in all 14 isolates (Fig. 3, Table 2). Seventeen additional proteins (just 11 % of the 632), including gamma-haemolysin components A and B and staphyloxanthin biosynthesis protein, were identified in 13/14 of the isolates. Overall, there appeared to be no threshold fraction that suggested that a proportion of the isolates might have a common proteome, regardless of lineage. In fact, the largest group comprised 144 (23 %) proteins that were detected in just a single isolate, thus demonstrating the extent of inter-isolate heterogeneity (Fig. 3). Interestingly, one Panton–Valentine leukocidin-positive strain, HHS-2, produced the lowest number of detectable exoproteins in total. This strain also produced the full range of recognized *S. aureus* exo-proteases (aureolysin; SspA/V8; SspB/staphopain B; ScpA/staphopain A; SplA–F). In contrast, none of the classical exo-proteases was detected from HHS-6, a strain that produced the greatest number of detected exoproteins.

**Table 2:** Proteins common to all 14 *S. aureus* strains Of the proteins identified, 52 occurred in all 14 isolates examined. It was possible to assign each protein to a COL sequence as indicated. The program psortb was used to determine the predicted cellular location (C, cytoplasmic; CM, cell membrane; CW, cell wall; E, exoprotein; U, unknown).

Reference	*S. aureus* protein name	Predicted location
SACOL0204	Formate acetyltransferase	C
SACOL0222	l-Lactate dehydrogenase	C
SACOL0452	Alkyl hydroperoxide reductase subunit C	C
SACOL0460	Inosine-5′-monophosphate dehydrogenase	C
SACOL0593	Elongation factor G	C
SACOL0594	Elongation factor Tu	C
SACOL0660	Alcohol dehydrogenase	C
SACOL0838	Glyceraldehyde 3-phosphate dehydrogenase	C
SACOL0839	Phosphoglycerate kinase	C
SACOL0842	Phosphopyruvate hydratase	C
SACOL1092	Phosphoenolpyruvate-protein phosphotransferase	C
SACOL1102	Pyruvate dehydrogenase complex E1 component, alpha subunit	C
SACOL1104	Branched-chain alpha-keto acid dehydrogenase	
subunit E2	C	
SACOL1105	Dihydrolipoamide dehydrogenase	C
SACOL1329	Glutamine synthetase FemC	C
SACOL1516	30S ribosomal protein S1	C
SACOL1637	Molecular chaperone DnaK	C
SACOL1722	Trigger factor	C
SACOL1729	Threonyl-tRNA synthetase	C
SACOL1745	Pyruvate kinase	C
SACOL1952	Ferritins family protein	C
SACOL2145	Glucosamine-fructose-6-phosphate aminotransferase	C
SACOL2213	DNA-directed RNA polymerase subunit alpha	C
SACOL2618	l-Lactate dehydrogenase	C
SACOL0545	50S ribosomal protein L25/general stress protein Ctc	C
SACOL2117	Fructose-bisphosphate aldolase	C
SACOL2224	50S ribosomal protein L6	C
SACOL2227	50S ribosomal protein L5	C
SACOL0778	Sulfatase family protein	CM
SACOL2002	Map protein, programmed frameshift	CM
SACOL0095	Immunoglobulin G binding protein A precursor	CW
SACOL0507	LysM domain-containing protein	CW
SACOL0609	sdrD protein	CW
SACOL0723	LysM domain-containing protein	CW
SACOL0856	Clumping factor A	CW
SACOL0317/0390	Lipase precursor, interruption-N	E
SACOL0860	Thermonuclease precursor	E
SACOL1062	Bifunctional autolysin	E
SACOL1173	Alpha-haemolysin precursor	E
SACOL2004	Leukocidin subunit precursor, putative	E
SACOL2006	Aerolysin/leukocidin family protein	E
SACOL2291	Staphyloxanthin biosynthesis protein	E
SACOL2421	Gamma haemolysin, component C	E
SACOL2584	Immunodominant antigen A	E
SACOL2660	Immunodominant antigen B	E
SACOL2666	*N*-Acetylmuramoyl-l-alanine amidase	E
SACOL2694	Lipase	E
SACOL1377	Transketolase	U
SACOL0962	Glycerophosphoryl diester phosphodiesterase GlpQ, putative	U
SACOL0303	5′-Nucleotidase	U
SACOL1704	Rod shape-determining protein MreC	U
SACOL2418	IgG-binding protein SBI	U

### Evidence of post-translational modifications

**Fig. 4:**
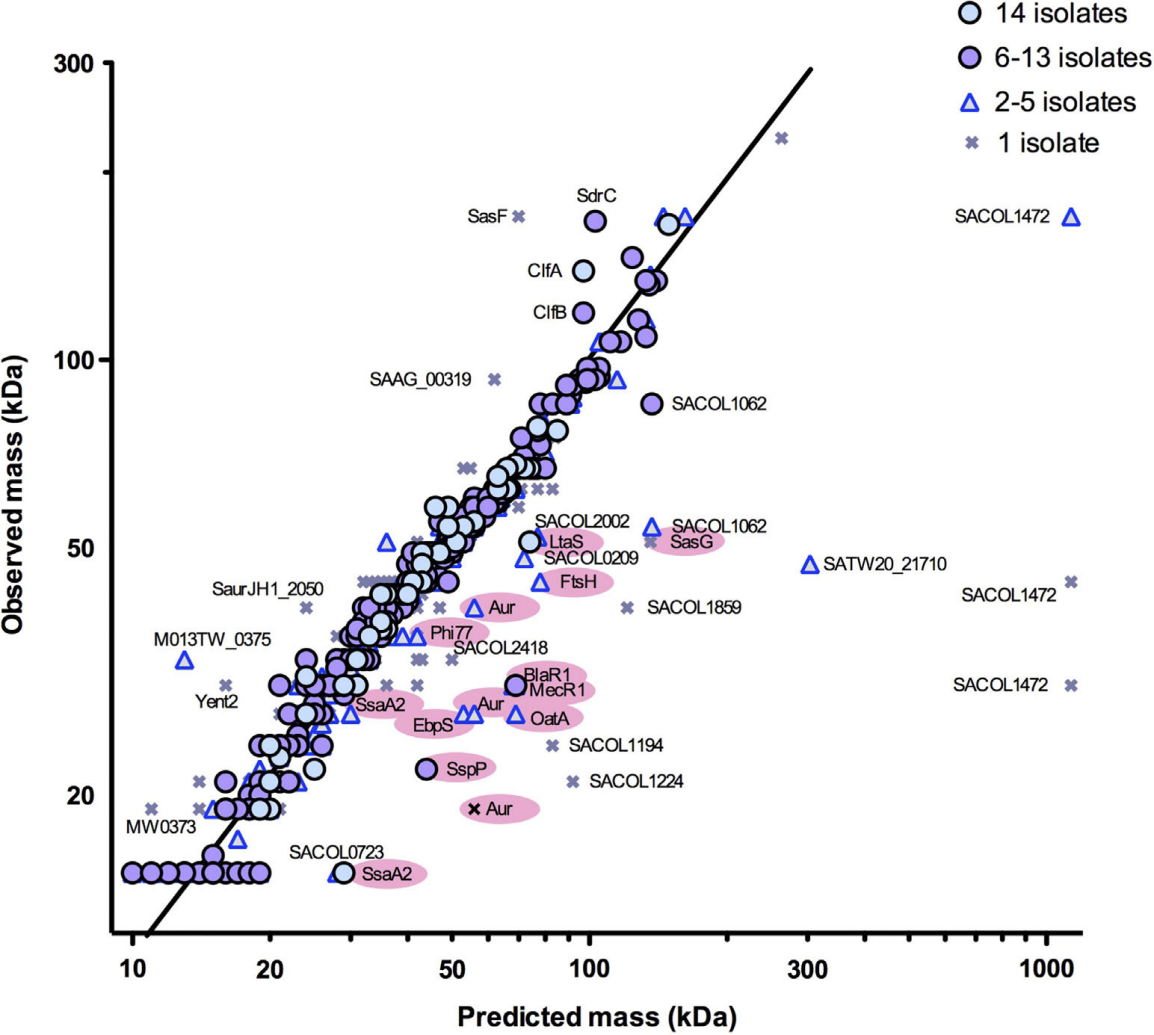
Comparison between the masses of *S. aureus* proteins predicted from amino acid sequences and masses observed from protein migration in SDS-PAGE. Proteins were identified in 1–14 of the *S. aureus* isolates studied; those identified to migrate at the mass predicted from amino acid sequences are plotted along the line of equivalence. Proteins to the left of the line were identified at masses greater than those predicted from the database. Proteins to the right of the line were identified at masses lower than those predicted and represent potential cleavage events or truncations. Data points have been overlaid such that those proteins found in the greater number of isolates are placed in the foreground. Proteins are labelled as SACOL-matching proteins or, where there is no match in COL, to another suitable reference database. Those proteins discussed in the text and referred to by short name codes are indicated as such and highlighted in pink.

**Fig. 5:**
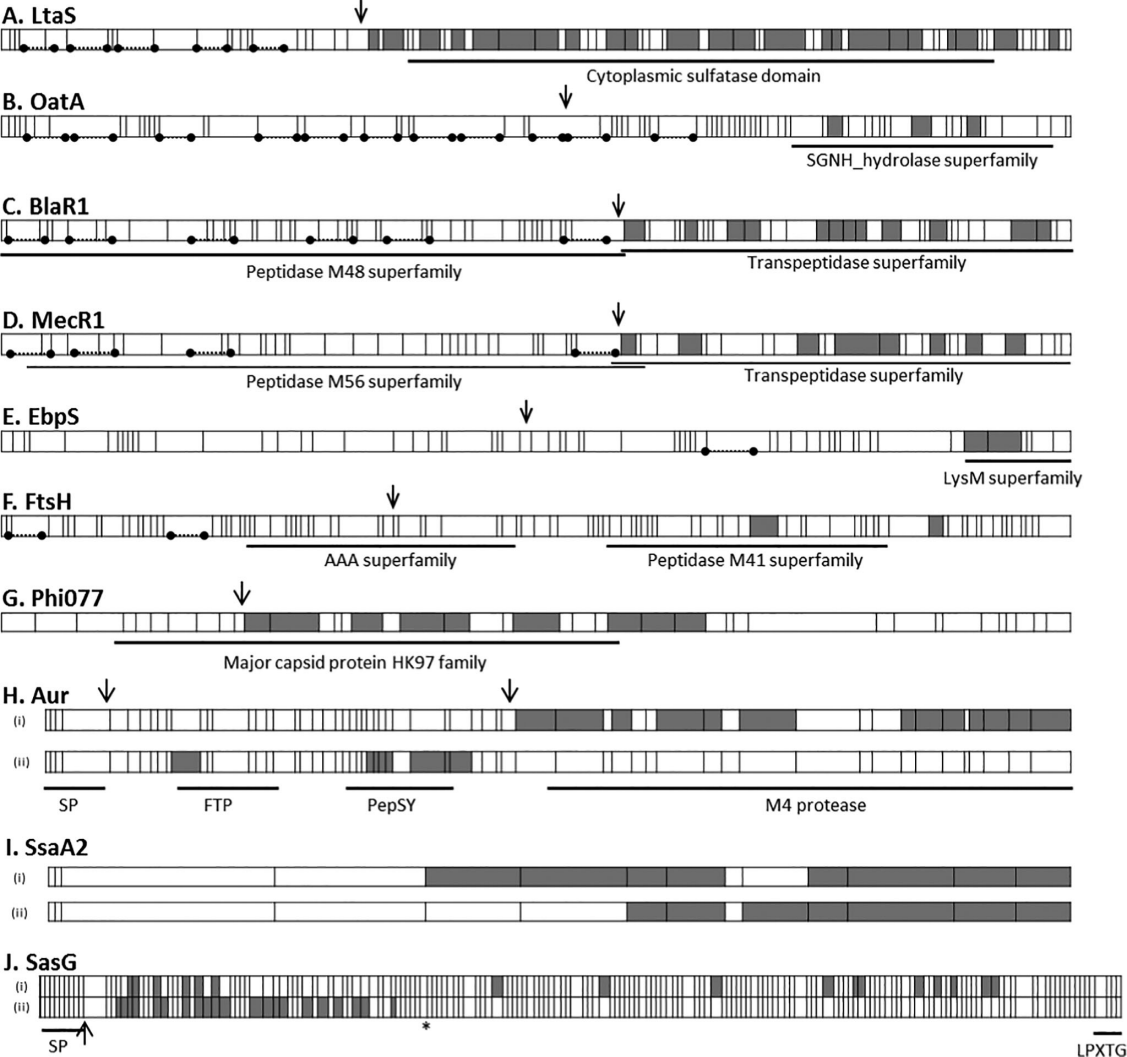
Structural characteristics and location of tryptic peptides of *S. aureus* proteins that migrated with lower than expected masses amongst the 14 isolates studied. Predicted trypsin cleavage sites (vertical bars) were determined using ExPASy cutter and those peptides detected by proteomics are shaded. Arrows indicate the position of known or predicted sites of proteolytic processing of the proteins. Transmembrane helices (predicted using the TMHMM server; http://www.cbs.dtu.dk/services/TMHMM/) are represented by dotted lines and filled circles. Known conserved domains and shown. SP, signal peptide. (A) LtaS (predicted mass: 74 kDa). Peptides corresponding to the C-terminal end only were identified in the gel region corresponding to a mass of ∼49 kDa in all 14 isolates examined. (B) OatA (predicted mass: 69 kDa). Peptides corresponding to the C-terminal end were identified in the ∼30 kDa gel region in isolates HHS-7, -11, -13 and -14. (C) BlaR1 (predicted mass: 69 kDa). Peptides corresponding to the C-terminal end were identified at ∼32 kDa in isolates HHS-2, -3, -4, -5, -8, -10 and -12. (D) MecR1 (predicted mass: 68 kDa). Peptides corresponding to the C-terminal end were identified at ∼32 kDa in isolates HHS-4 and -11. (E) EbpS (predicted mass: 53 kDa). Peptides corresponding to the C-terminal end were identified at ∼25 kDa in isolates HHS-1, -3, -5 and -13. (F) FtsH (predicted mass: 78 kDa). Peptides corresponding to the C-terminal end were identified at ∼45 kDa in isolates HHS-9, -10 and -13. (G) Phi077 ORF006-like protein (predicted mass: 42 kDa). Peptides corresponding to the central region of the protein were identified at ∼32 kDa in isolates HHS-4, -6, -9 and -11. (H) Aur (predicted mass: 56 kDa). Peptides were located in two regions: in one (i) at ∼40 kDa peptides corresponding to the C-terminal end were identified in isolates HHS-2, -4, -12 and -13, whereas in the other (ii) at ∼25 kDa peptides corresponding to the N terminus were found in isolates HHS-12 and -13. (I) SsaA2 (predicted mass: 30 kDa). Peptides were identified in all 14 isolates in two gel regions at (i) ∼30 kDa and (ii) ∼15 kDa. (J) SasG (predicted mass: 136 kDa). Peptides were identified in two gel regions at (i) ∼140 kDa in isolates HHS-1, -3 and -8 and (ii) ∼36 kDa in isolate HHS-5.*Position of premature stop codon in *SasG* gene.

As the supernatant proteins were first separated by SDS-PAGE prior to proteomic identification, it was possible to estimate approximate masses from their migration and to compare these values with those expected from the predicted sequences. A plot of these data showed that 95 % of the proteins identified migrated as expected (Fig. 4). However, about 1 % of the proteins did not migrate as far through the gel as expected and hence appeared to have a larger mass than predicted from their sequence. These included serine-aspartate repeat-containing protein C (SdrC), clumping factors A and B (ClfA, ClfB), putative surface anchored protein (SasF), SAAG_00319 (fibronectin binding protein B), SaurJH1_2050 (phi13 family phage major tail protein), SAV1826 (enterotoxin, Yent2) and several hypothetical proteins. The remaining 4 % of the proteins identified migrated faster than expected, i.e. they appeared to have smaller masses than predicted. Particularly marked differences were apparent for LtaS, OatA, BlaR1, MecR1, EbpS and FtsH (Fig. 4). Several proteins were found to be represented as two or more different masses, including SsaA2, Aur, SspP and extracellular matrix-binding protein (Ebh) (SACOL1472). Such migration is consistent with proteolytic cleavage. To investigate this further, the distribution of the peptides detected within the protein sequences was examined and, in 11 cases, there was sufficient peptide information to be informative (Fig. 5). For LtaS, OatA, BlaR1, MecR1 and EbpS, the peptides were located in the C-terminal region of each protein (Fig. 5) suggesting a post-translational cleavage of the N terminus in each case. SsaA2 appeared to be present as both a full-length protein and a cleaved product of ∼15 kDa in all 14 isolates. Unusually, amongst all the peptides identified, one peptide was only detected in the cleaved product (12/14 isolates) but was not detected in the uncleaved precursor (all 14 isolates). Peptides corresponding to Phi77 ORF006-like protein were located together in a region that corresponds to a protein mass of 32 kDa, as observed. Aur was detected as both a 43 and 25 kDa form. The 43 kDa form contained peptides from the C-terminal region only whereas the 25 kDa form contained peptides only located in the N-terminal region of the protein.

SasG was found in three isolates where it migrated according to its predicted mass of 136 kDa, although in strain HHS-5 it ran as a 49 kDa protein, identified from peptides occurring in the N-terminal region only (Fig. 5H). This might be explained by a nucleotide deletion at nucleotide 1287 of *sasG*, which is known to occur in several clinical isolates although a truncated protein has not previously been identified (Diep *et al.*, 2006; Geoghegan *et al.*, 2010). Consequently, PCR amplification and sequencing of the central region of the *sasG* gene from isolate HHS-5 was undertaken. Comparison of the sequence data with the COL genome showed a single nucleotide deletion 100 nt downstream of the 5′ primer (COL: AAAAAAAGTT; HHS-5: AAAAAA-GTT) resulting in a premature stop codon.

## Discussion

The present study set out to characterize the exoproteome of representatives of dominant clones of *S. aureus* that occur in the UK, specifically to identify common core exoproteins. However, despite 70 % of *S. aureus* genes being considered to belong to the core genome, only 8 % of the proteins detected were shared among these clinically relevant strains. Furthermore, some 60 % of the proteins identified were not ‘professional’ exoproteins, but were of predicted cytosolic and metabolic origin. Finally, it was possible to clearly identify cleaved and truncated protein products simply from proteomic comparison of observed and expected molecular masses.

Previous studies have identified up to 250 different exoproteins in *S. aureus* culture supernatants (Burlak *et al.*, 2007; Pocsfalvi *et al.*, 2008), while, in this present analysis we found a total of 632 proteins, with over 400 in some individual strains. The greater number of proteins identified is likely to result from the number of strains evaluated, use of a more sensitive 1D in-gel analytical technique, and the use of a non-repetitive database of manageable size that aimed to link each identified protein to a single reference strain only. The result of the analysis performed in this way is inclusive, as it is not based on comparison with any one genome/predicted proteome and is easier to interpret as it is referenced principally to one proteome. In this case, we chose to use *S. aureus* COL as the reference database, as the characteristics of this strain are well documented, but the whole process could just as easily be based on any suitable reference database. As the approach makes comparisons between the protein sequences, difficulties with inconsistent or incorrect annotations that occur between sequence databases are avoided.

Overall, there was considerable heterogeneity between the proteins identified amongst the 14 isolates analysed in this study, as has been reported previously for *S. aureus* (Ziebandt *et al.*, 2010). The clinical strains were selected to be representative of UK clinical isolates and to be genetically diverse, as confirmed by subsequent whole genome sequencing (Fig. S2). Although the proteomic study was conducted once only, there were no apparent lineage-specific features in the proteins identified, apart from genes that are variably present.

While Ziebandt *et al.* (2010) reported just seven Sec-dependent extracellular proteins in 17 clonally different strains that they examined, our study identified 52 core extracellular proteins, although several proteins reported therein were also identified in our study incuding IsaA. Of the 52 common proteins detected in the current study, psortb predicted a cytoplasmic localization for 28 of them. The appearance of cytoplasmic proteins is common in studies of the secretome (Ebner *et al.*, 2015; Foulston *et al.*, 2014; Henderson & Martin, 2011; Sibbald *et al.*, 2006; Tjalsma *et al.*, 2004). Release of cytosolic proteins could be related to *S. aureus* production of membrane vesicles or other specific efflux mechanisms (Ebner *et al.*, 2015; Lee *et al.*, 2009), although the activity of bifunctional autolysin (Atl) and amidase (SACOL2666), which were detected in all strains, and the necessary process of cell-wall remodelling during growth cannot be discounted.

Post-translational modification of several proteins was apparent, as evident from aberrant migration or the appearance of multiple bands following SDS-PAGE. Some of these proteins migrated more slowly than estimated from their predicted structure, suggesting a greater mass than expected. Several of these proteins, including serine-aspartate repeat-containing protein C (SdrC), clumping factors A and B (ClfA, ClfB), and putative surface anchored protein (SasF), are known, or presumed, to be post-translationally modified through a covalent anchor to the peptidoglycan of the cell-wall envelope, which would explain their increased mass. The other outliers include SAAG_00319 (fibronectin binding protein B), SaurJH1_2050 (phi13 family phage major tail protein), SAV1826 (enterotoxin, Yent2) and several hypothetical proteins for which amino acid composition or multimerization may explain aberrant migration.

A greater number of proteins was found that migrated more rapidly than estimated from their amino acid structure. Some were evident in multiple rows, suggesting proteolysis resulting in cleavage products with smaller masses compared with the parent proteins. The majority of rapidly migrating proteins represented known mature products that had resulted from defined proteolytic processes rather than as the result of unspecific proteolytic digestion as might occur if the cells were extensively lysed, underlining the value of comparing actual and predicted molecular size when undertaking proteomic studies. There is evidence from several previous studies to support the assertion of N-terminal cleavage of LtaS (Gatlin *et al.*, 2006; Gründling & Schneewind, 2007; Wörmann *et al.*, 2011), OatA (Schallenberger *et al.*, 2012), BlaR1 (Llarrull *et al.*, 2011; Powers *et al.*, 2011; Zhang *et al.*, 2001), EbpS (Downer *et al.*, 2002; Nakakido *et al.*, 2007; Park *et al.*, 1991, 1996), FtsH (Akiyama, 1999; Karnataki *et al.*, 2009; Krzywda *et al.*, 2002) and Phi77 ORF006-like protein (Conway *et al.*, 1995; Duda *et al.*, 1995). Similarly, Aur is known to undergo sequential processing resulting in two mature products (Nickerson *et al.*, 2008). SsaA2 was found to be present as both a full-length protein and an N-terminally cleaved product of ∼15 kDa in all 14 isolates. To our knowledge, this does not appear to have been described previously, demonstrating the added value of molecular size comparisons in proteomic studies.

The surface protein SasG was found in the culture supernatant of just three isolates, where it migrated according to its predicted mass of 136 kDa. In HHS-5, however, which represents a USA300-like CC8 lineage, the gene exhibits a premature stop codon. Accordingly, SasG ran as a 49 kDa protein, identified from peptides occurring in the N-terminal region only, corresponding to the A domain of a truncated SasG. To our knowledge, this is the first evidence that the A domain is expressed, secreted and stable enough to be detected when SasG is truncated. Studies point to a role of SasG in *S. aureus* biofilm formation, attributed largely to the bacterial cell-wall-bound B domain of the protein (Corrigan *et al.*, 2007; Roche *et al.*, 2003). Although a specific role for soluble A domain has yet to be proven, the finding may be of importance given that recombinant A domain can contribute to auto-aggregation through homo-oligomerization (Kuroda *et al.*, 2008).

Identification of the core exoproteins produced in broth that are common to clinically relevant *S. aureus* lineages may provide potential immunodiagnostic biomarkers of *S. aureus* growth, for example in blood cultures, where rapid distinction from coagulase-negative staphylococci would be advantageous. Biomarkers could include those with housekeeping functions, provided that there is no orthologue in other staphylococci, or provided that the C-terminal sequence is unique, making them suitable targets for C-terminal antibody-based diagnostics (Edwards *et al.*, 2007). Potential candidates include alcohol dehydrogenase, both lactate dehydrogenases and staphylococcal immunodominant antigen A (IsaA), although these findings cannot be extrapolated to clinical samples produced *in vivo* without further experimentation.

In conclusion, we have determined the experimental exoproteome of the important human pathogen *S. aureus* in 14 genetically diverse, clinically relevant isolates of the species. As well as identifying the presence of a variety of functionally relevant proteins we have also used this large dataset to reveal information concerning protein processing and truncation of SasG. Our approach was unbiased, and did not place emphasis on any particular class of protein, such as virulence factors, nor make assumptions about which proteins should or should not be found in the supernatant. Indeed, many proteins found outside the cell did not have predictable signal sequences and, whilst they may be products of cell turnover, it is also possible that they are performing as yet unknown functions on the surface or outside the cell.

## Supplementary Data

116 Supplementary Data
