# Supplementary material for: Identification of commonly expressed exoproteins and proteolytic cleavage events by proteomic mining of clinically relevant UK isolates of Staphylococcus aureus
Source: Microb Genom. 2016 Feb 23;2(2):e000049. doi: 10.1099/mgen.0.000049 (PMC5320583; doi:10.1099/mgen.0.000049)

Supplementary Material

Supplementary Figure 1. *Individual strain growth curves.* Optical density (OD_{600}) against time is provided for strains HHS-1 – HHS-14. Error bars show standard error of mean of three separate analyses on different days in fresh LB.

Supplementary Figure 2. *Genomic SNP comparison of strains HHS-1 – HHS-14.* Strains underwent whole genome sequencing (WGS) on an Illumina HiSeq Instrument. Sequence reads were mapped with bowtie2 against MLST loci. The genetic relatedness of the 14 strains was assessed by mapping sequence reads to the published ST22 genome H050960412 using BWA(0.7.5). Single Nucleotide Polymorphisms (SNP) were called using GATK2.6.5. Genetic relatedness was determined using only high quality SNPs (AD genotype = 0.9). SNPs were concatenated and aligned (allowing Ns in 0.4 frequency) and analysed by Maximum Likelihood (MEGA 6). Phylogeny was inferred by Maximum Likelihood method (Jukes-Cantor model) with bootstrap (n=1000) analysis. The tree is drawn to scale, with branch lengths measured in the number of substitutions per site. There were a total of 28796 positions in the final dataset. Red triangle indicates reference genome.

Supplementary Table 1 (255kb PDF file) *S. aureus proteins identified in strains HHS-1 – HHS-14.* Protein identifications by protein ID. Col was used as a primary reference. All non-COL hits were linked to a COL reference where possible (as described in Materials & Methods section), as these probably indicate that an allelic variant was present rather than different proteins. Identifications from peptides comprising highly similar modular proteins or abundant contaminating proteins should be interpreted with caution and

with reference to Table 1 and genome data. Numbers indicate the row that protein was excised from.

Supplementary Table 2. (184kb PDF file) The identity of each of the *S. aureus* proteins in strains HHS-1 – HHS-14 that are listed in in Supplementary Table 1 was supported by determination of the statistical probability of the identification in each case using the SEQUEST algorithm. Numbers in the table represent probability in each strain, except for those where the corresponding protein was not detected (-).

Supplementary Table 3. (1.8MB PDF file) Identification of the *S. aureus* proteins in strains HHS-1 – HHS-14 listed in Supplementary Tables 2 was underpinned by mass spectrometric matches of ions to tryptic peptides. For each peptide found in the corresponding sample from each strain, its primary structure, charge and derived XCorr value is shown, except for those where no corresponding peptide was detected (-). Where the same peptide was found repetitively in the same strain the XCorr value with greatest certainty is shown.

Supplementary Table 4 (3.5MB text file) Non-overlapping combined database used to identify *S. aureus* proteins. Database comprises 31804 proteins of which 31784 are non-identical *S. aureus* proteins plus commonly found contaminants – inclusion of which ensures contaminants are identified as contaminants i.e. removes possibility that MS data generate false positive identifications.

Supplementary Figure 1

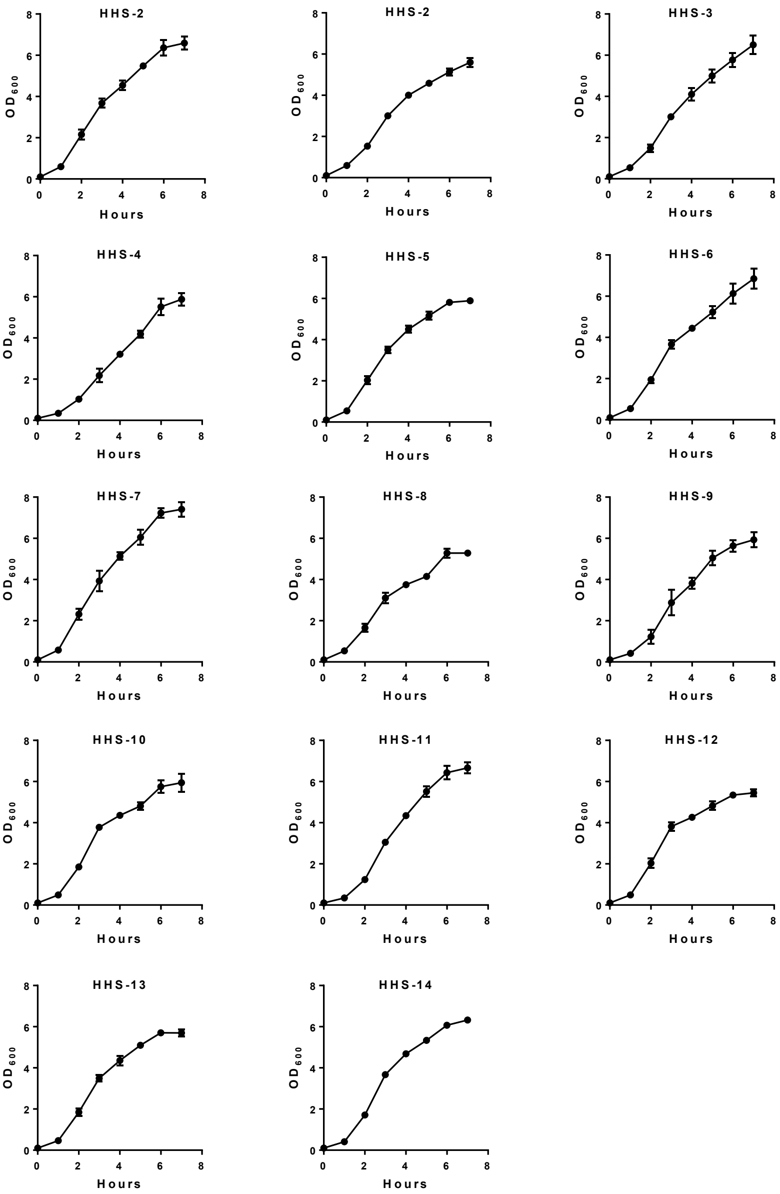

Supplementary Figure 2

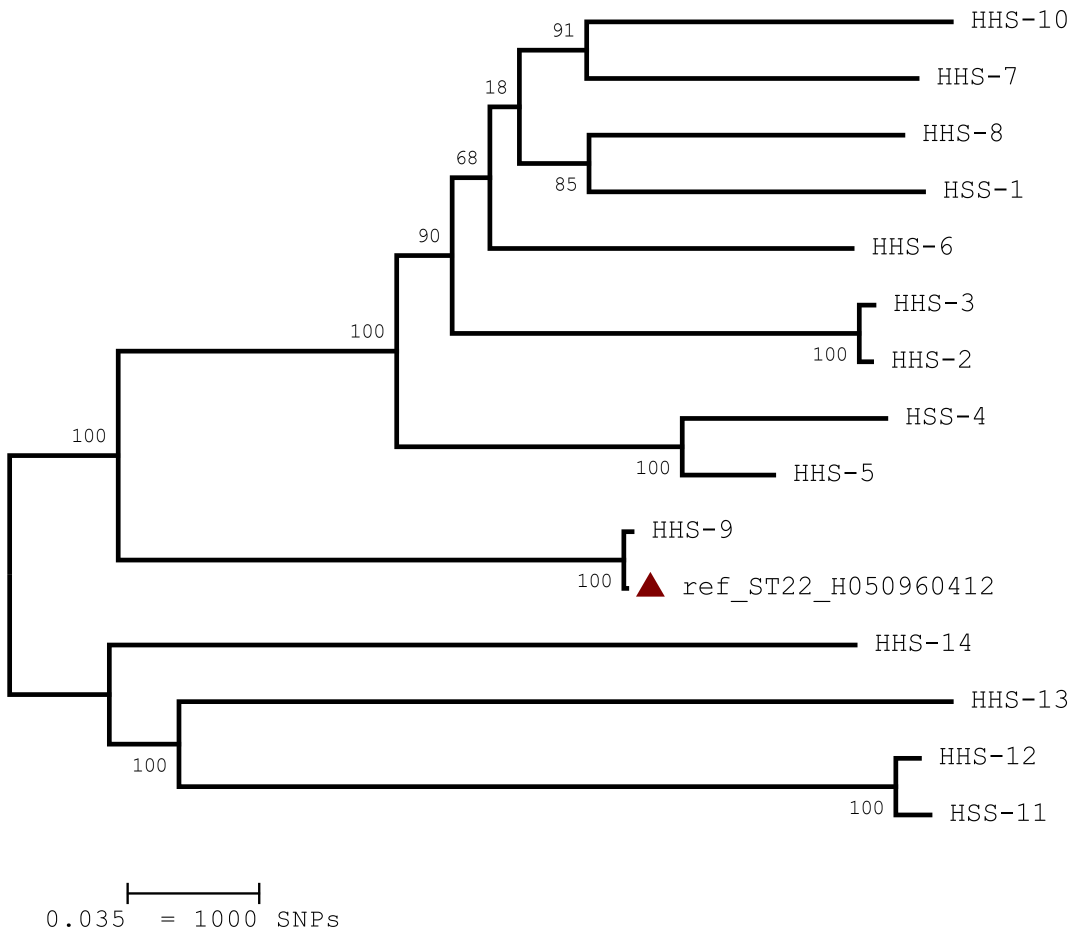

Supplement: Supplementary file 1 — Supplementary Data [file mgen-02-49-s001.pdf]
